# Archaic chaos: intrinsically disordered proteins in Archaea

**DOI:** 10.1186/1752-0509-4-S1-S1

**Published:** 2010-05-28

**Authors:** Bin Xue, Robert W Williams, Christopher J Oldfield, A Keith Dunker, Vladimir N Uversky

**Affiliations:** 1 Center for Computational Biology and Bioinformatics, Indiana University School of Medicine, Indianapolis, IN 46202, USA; 2 Institute for Intrinsically Disordered Protein Research, Indiana University School of Medicine, Indianapolis, IN 46202, USA; 3 Department of Biomedical Informatics, Uniformed Services University, Bethesda, MD 20814 , USA; 4 Center for Computational Biology and Bioinformatics, Indiana University School of Informatics, Indianapolis, IN 46202, USA; 5 Institute for Biological Instrumentation, Russian Academy of Sciences, 142290 Pushchino, Moscow Region, Russia

## Abstract

**Background:**

Many proteins or their regions known as intrinsically disordered proteins (IDPs) and intrinsically disordered regions (IDRs) lack unique 3D structure in their native states under physiological conditions yet fulfill key biological functions. Earlier bioinformatics studies showed that IDPs and IDRs are highly abundant in different proteomes and carry out mostly regulatory functions related to molecular recognition and signal transduction. Archaea belong to an intriguing domain of life whose members, being microbes, are characterized by a unique mosaic-like combination of bacterial and eukaryotic properties and include inhabitants of some of the most extreme environments on the planet. With the expansion of the archaea genome data (more than fifty archaea species from five different phyla are known now), and with recent improvements in the accuracy of intrinsic disorder prediction, it is time to re-examine the abundance of IDPs and IDRs in the archaea domain.

**Results:**

The abundance of IDPs and IDRs in 53 archaea species is analyzed. The amino acid composition profiles of these species are generally quite different from each other. The disordered content is highly species-dependent. **Thermoproteales** proteomes have 14% of disordered residues, while in **Halobacteria**, this value increases to 34%. In proteomes of these two phyla, proteins containing long disordered regions account for 12% and 46%, whereas 4% and 26% their proteins are wholly disordered. These three measures of disorder content are linearly correlated with each other at the genome level. There is a weak correlation between the environmental factors (such as salinity, pH and temperature of the habitats) and the abundance of intrinsic disorder in Archaea, with various environmental factors possessing different disorder-promoting strengths. Harsh environmental conditions, especially those combining several hostile factors, clearly favor increased disorder content. Intrinsic disorder is highly abundant in functional Pfam domains of the archaea origin. The analysis based on the disordered content and phylogenetic tree indicated diverse evolution of intrinsic disorder among various classes and species of Archaea.

**Conclusions:**

Archaea proteins are rich in intrinsic disorder. Some of these IDPs and IDRs likely evolve to help archaea to accommodate to their hostile habitats. Other archaean IDPs and IDRs possess crucial biological functions similar to those of the bacterial and eukaryotic IDPs/IDRs.

## Introduction

### Introducing Archaea

It is known that all the living systems on the Earth can be divided into three large domains, the Bacteria, the Archaea, and the Eucarya, each containing at least two kingdoms [1-3]. The Bacteria and the Archaea domains include single-celled microorganisms, prokaryotes. Although archaea are similar to bacteria phenotypically (both have no cell nucleus or any other cellular organelles inside their cells and are very often similar in size and shape), and despite a bacterial organization of archeae chromosome (messenger RNA with Shine-Dalgarno sequences, genes assembled in operons, a single origin of bidirectional replication), these two domains of life are clearly different at the molecular level, and some of the archaea genes, metabolic pathways and proteins (especially ribosomal proteins and proteins involved in transcriptions and translation) are more closely related to those of eukaryotes [4-11]. For example, all eubacteria exhibit very similar subunit pattern in their RNA polymerases (in terms of numbers and sizes), whereas this pattern is not related to that seen in the archaea or the eukaryotes [4], and several archaea and eukaryotic ribosomal protein homologues have no apparent counterpart among the bacteria [5,6]. On the other hand, archaea and eukaryotes are sufficiently dissimilar and diverged early, and, therefore, they could not be placed in a single domain of life either [1]. Generally speaking, according to the detailed molecular analysis and comparative genomics, archaea are characterized by a combination of unique properties, such as left-handed isoprenoids containing glycerolipids, and mosaic bacterial and eukaryotic features [12].

Based on sequences of ribosomal RNAs, archaea were first classified as a separate group of prokaryotes in 1977 [13]. Before that time prokaryotes were considered as a single group. The term “archaea” was introduced in 1987 to denote apparent primitive nature of corresponding organisms especially in comparison with the eukaryotes [2]. It is estimated that the total number of phyla in the archaea domain range from 18 to 23, of which only 8 phyla have representatives that have been grown in culture and studied directly [14]. In fact, most of the culturable and well-investigated species of archaea belong to the two main phyla, **Crenarchaeota**, and **Euryarchaeota**. Three new phyla, **Thaumarchaeota**, **Nanoarchaeota**, and **Korarchaeota**, were discovered very recently. **Nanoarchaeota** contains a nanosized symbiotic hyperthermophilic archaeon *Nanoarchaeum equitans* from a submarine hot vent, which grows attached to the surface of a specific archaeal host, a new member of the genus ***Ignicoccus***[15]. Based on the small subunit rRNA phylogeny it has been concluded that** Korarchaeota** comprises a group of microorganisms that may have diverged early from the major archaeal phyla **Crenarchaeota** and **Euryarchaeota**, share many features of both of these main phyla, but are most closely related to the **Crenarchaeota**[16]. Members of the **Thaumarchaeota** phylum are mesophilic archaea which are different from hyperthermophilic **Crenarchaeota** to which they were originally ascribed [17].

It is recognized now that archaea are an important component of the biosphere [11], play important roles in the carbon and nitrogen cycle, and may contribute up to 20% of the total biomass on Earth [18]. The unique feature of some archaea is their ability to produce methane gas in anaerobic environments; i.e., methanogenesis. Another uniqueness of the archaea is their ability to utilize a great variety of energy sources ranging from sugars, to using ammonia, sulfur, metal ions and even hydrogen gas as nutrients; some salt-tolerant archaea (the ***Halobacteria***) use sunlight as a source of energy; other archaea use CO_2_ in the atmosphere as a source of carbon via the carbon-fixation process, which is powered by inorganic sources of energy, rather than by capturing sunlight [19-21]. Many archaea are able to grow at temperatures above 100^o^C and are found in geysers, black smokers, and oil wells. The archaeon *Methanopyrus kandleri* (Strain 116) can effectively grow at 122°C and high hydrostatic pressure (20 MPa), which is the highest recorded temperature at which an organism will grow [22]. Others are found in very cold habitats and still others can survive in highly saline, acidic (at pHs as low as 0, which is equivalent to 1.2 M sulfuric acid), or alkaline water [23]. In addition to these extremophiles (halophiles, hyperthermophiles, thermophiles, psychrophiles, alkaliphiles, and acidophiles), many archaea are mesophiles that grow in much milder conditions, such as marshland, sewage, the oceans, and soils [24]. Although for a long time Archaea, in particular **Crenarchaeota**, were considered ecologically insignificant, presuming to occupy mainly extreme and unusual environments, it is becoming increasingly evident that previously unrecognized members of the Archaea are abundant, globally distributed, and well-adapted to more pedestrian lifestyles and niches, including symbiotic partnership with eukaryotic hosts [25]. Archaea are particularly numerous in the oceans, and the archaea in plankton (as part of the picoplankton) may be one of the most abundant groups of organisms on the planet, accounting for up to 40% of the bacterioplankton in deep ocean waters [26]. Therefore, it has been pointed out that the study of archaea is essential to understand the history of molecular mechanisms and metabolism diversity and to unravel the mechanisms by which life can sustain in extreme environments [12].

### Introducing intrinsically disordered proteins

As verified by an increasing number of experimental observations, more and more proteins or their regions have been found to lack unique 3D structure in their native states under physiological conditions. These regions and proteins, known as Intrinsically Disordered Regions (IDR) or Intrinsically Disordered Proteins (IDP) among different other names [27-30], present in solution as conformational ensembles containing large number of widely different conformations that are in rapid interconversion on different time scales. The protein intrinsic disorder phenomenon is rapidly becoming well-accepted in modern protein science. Unlike structured proteins, IDPs stay as an ensemble of flexible conformations [27,31-33]. Although without stable 3D structures and in contradiction to the traditional sequence-structure-function paradigm, IDPs play a number of crucial functional roles in living organisms, especially in vital biological processes, such as signaling, recognition, and regulation [27,31,32]. According to a statistical study on SwissProt database, 238 out of 710 SwissProt functional keywords are strongly positively correlated with intrinsic disorder, while another 302 functional keywords mostly characterizing various catalytic activities are strongly negatively correlated with IDR [34].

Due to their crucial functional roles, IDPs are highly abundant in all species. According to computational predictions by PONDR^®^-VLXT, typically 7-30% prokaryotic proteins contain long disordered regions of more than 30 consecutive residues, whereas in eukaryotes the amount of such proteins reaches 45-50% [28,35-38]. Another estimation based on DISOPRED2 achieved similar results: around 2.0%, 4.2%, and 33.0% of proteins in archaea, bacteria, and eukaryota have long disordered segments with 30 or more residues [39]. Higher contents of long IDR were reported in a study using another computational tool, DisEMBL [40]. In that study, 23~56%, 15~40%, and 25~78% of proteins in archaea, bacteria, and eukaryota were predicted to have IDR longer than 40 residues. In spite of the disagreement between the reported values, the general trend among the three domains of life is quite consistent: at the proteome level, eukaryotes have much more disordered proteins than bacteria and archaea. This is a reflection of the vital roles of IDPs and IDRs in signaling and regulation. Furthermore, not only at proteome level, but even in PDB, which is biased to structured proteins, intrinsic disorder is also very abundant, and almost 70% of proteins in PDB have IDRs which are indicated by missing electron density [41].

Despite of the solid proofs of the relative abundance of IDPs in nature, their origin is still a mystery. Where are they coming from? How do they evolve? Although all of the three domains of life have a considerable amount of intrinsic disorder, modern species have evolved so effectively that ancient information is no longer easy to retrieve. In this meaning, archaea could be an excellent candidate to tell the story of what happened thousands of millions years ago. Since archaea are prokaryotes (they have no cell nucleus or any other organelles within the cell), they seem to have appeared early in the evolution. Furthermore, many archaea live and grow at extreme conditions, such as high temperature, which are believed to be very similar to the conditions at the early time of planet formation. Finally, archaea have genes and several metabolic pathways which are more similar to eukaryotes than bacteria. Hence, by taking into account the facts that eukaryotes need more signaling and regulation due to their biological complexity, and that eukaryotes are highly enriched in IDRs and IDPs, archaea may provide interesting information about the evolution of intrinsic disorder.

Previous studies discussed above provided very enlightening information on the abundance of intrinsic disorder in archaea. However, at that time the number of species available for the bioinformatics analysis was rather limited. Studies utilizing PONDR^®^-VLXT, DISOPRED2, and DisEMBL had only 7, 6, and 20 archaea species, respectively [39,40,42]. This limited number of species restricted the study on the phylogenetic relations among the archaea species. Hence, with the expansion of archaea genome data (more than fifty archaea species from five different phyla are known now), it is necessary to re-examine the previous results and to explore new information. Here, we systematically studied the abundance of intrinsic disorder in archaea and explored the functional and evolutionary roles of intrinsic disorder in this domain of life.

## Methods

### Datasets

All protein sequences from the completed 53 archaea genome were downloaded from the ExPASy proteomics server as of Jan. 2009 [43]. The taxonomy of these archaea is listed in Table S1 (see additional file 1). Note: In the following discussion, names of phyla are in **bold**; names of classes and orders are in ***bold italic***; whereas names of species are in *italic*. All five known phyla of archaea are included in this study: **Crenarchaeota** and **Euryarchaeota** have 15 and 32 species, respectively, each of the **Thaumarchaeota** and **Nanoarchaeota** phyla has two species; and finally there is only one species in the **Korarchaeota** phyla. All the species in **Korarchaeota**, **Thaumarchaeota**, and **Nanoarchaeota** can be grouped into one class corresponding to that phylum. Although **Crenarchaeota** has 15 species, all of these species also belong to a single class, ***Thermoprotei***. Hence, these species could be combined together and be analyzed as a single one. **Euryarchaeota** is the most complicated phylum of archaea. It has 7 classes with one to twelve species in each of them. In order to take this complexity into consideration, following analysis will be conducted at three different levels: 5 phyla, 11 classes, and 53 species.

### Disorder predictions

In this study, two types of intrinsic disorder predictors were utilized, per-residue predictors and binary classifiers. Per-residue predictors provide the distribution of the propensity for intrinsic disorder over the amino acid sequence, whereas binary classifiers identify entire protein as wholly ordered or wholly disordered. The per-residue predictors were used to generate two means for the evaluation of abundance of intrinsic disorder in a given protein, the total amount of disordered residues and the number of long disordered regions containing >30 consecutive amino acid residues predicted to be disordered. The binary classifiers were used to evaluate the number of wholly disordered proteins in a given proteome.

#### Per-residue disorder predictions

**Figure 1:**
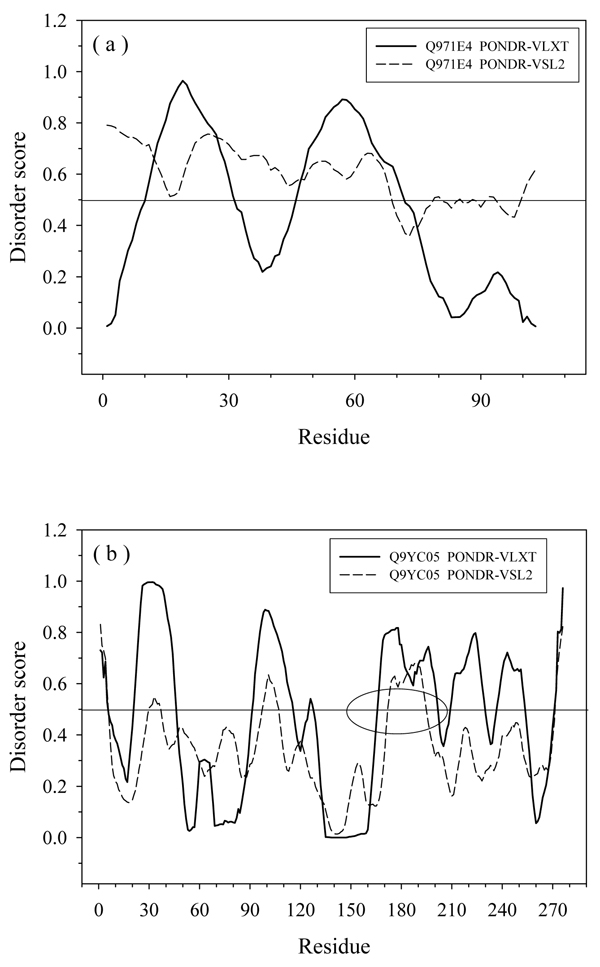
**Comparison of disorder prediction between PONDR-VLXT and PONDR-VSL2 for (a) Q971E4 and (b) Q9YC05:** The solid line is the disorder score of PONDR-VLXT, while the dashed line is from PONDR-VSL2. The line at (a) shows a dip in VLXT prediction while VSL2 predicts the long segment to be disordered. The circle in (b) represents a long disordered region predicted by VLXT, but missed by VSL2.

In this study, per-residue disorder predictors PONDR^®^-VLXT [36] and PONDR^®^-VSL2 [44] were utilized. PONDR^®^-VLXT is the first disorder predictor which was designed by using neural networks. It is very sensitive to the changes of local compositional profile. One of its prominent properties is the frequently occurring dips on the plot of disorder score (see Figure 1). These dips correspond to hydrophobic segments with the increased propensity to order that are flanked by disordered regions. many of these segments are found to be very important in molecular recognition, signaling and regulation. They are now recognized as a Molecular Recognition Feature (MoRF) [38,45]. PONDR^®^-VSL2 is composed of a set of support vector machines and was trained on datasets containing disordered regions of various lengths. It is one of the most accurate predictors developed so far. Both PONDR^®^-VLXT and VSL2 have been applied in genome-wide studies on protein intrinsic disorder. The results of these analyses clearly indicated the existence of noticeable differences between these two predictors. However, the sources of these differences and their underlying biological significance have not been clearly uncovered as of yet. Figure 1 represents the illustrative example of the disorder evaluation by PONDR^®^-VLXT and PONDR^®^-VSL2 predictors in two unrelated proteins. This figure illustrates the typical feature of the PONDR^®^-VLXT plot which contains many sharp dips. As a result, long disordered regions are divided into a series of short disordered regions by these dips. Consequently, PONDR^®^-VLXT may under-estimate the ratio of long disordered regions as shown in Figure 1(a). On the other hand, although PONDR^®^-VSL2 is more accurate than PONDR^®^-VLXT on short disordered/structured regions, it was also trained using a set of short protein segments. As a result, for proteins that tend to have intersected disordered/structured segments, PONDR^®^-VSL2 may also have lower ratio for long disordered regions as indicated by Figure 1(b). Hence, it would be beneficial to combine the results of several different predictors. However, in this study, due to reasons discussed above, we will focus on the results from the PONDR^®^-VSL2.

#### Binary disorder classification

Based on the per-residue disorder prediction, a Cumulative Distribution Function (CDF) can be obtained to describe the disorder status of the entire protein [37,42,46]. Basically, CDF is based on a cumulated histogram of disordered residues at various disorder scores. By definition, structured proteins will have more structured residues and less disordered residues. Therefore, the CDF curve of a structured protein will increase very quickly on the side of low disorder score, and then go flat on the side of high disorder score. On the other hand, for disordered proteins, the CDF curve will move upward slightly in regions of low disorder score, then rapidly increase in the regions with high disorder scores. Hence, on the 2D CDF plot, structured proteins tend to be located in the upper left half, whereas disordered proteins are predominantly located at the lower right half of the plot. By comparing the locations of CDF curves for a group of fully disordered and fully structured proteins, a boundary line between these two groups of proteins can be identified. Then, this boundary can be used to classify any given protein as wholly ordered or wholly disordered. Proteins whose CDF curves are above the boundary line are mostly structured, whereas proteins with CDF curves located below the boundary are mostly disordered [37,42,46]. The distance of a curve from the CDF boundary can also be used as a kind of measure of the disordered (structured) status of a protein. This distance is further referred as CDF-distance. Originally, CDF analysis was developed based on the results of the PONDR^®^-VLXT [28]. Recently, other five CDF predictors were built using the outputs of the PONDR^®^-VSL2 [44], PONDR^®^-VL3 [47], IUPred [48], FoldIndex [49], and TopIDP [50]. Among these various CDFs, PONDR^®^-VSL2-CDF achieved the highest accuracy, 5-10% higher than the accuracy of the second best predictor [46].

Another method of measuring the disordered status of the entire protein is a Charge-Hydropathy (CH) plot [29]. CH-plot takes the averaged Kyte-Doolittle hydrophobicity [51] and an absolute mean net charge of a protein chain as the coordinates of the X- and Y-axis, respectively. This plot represents each protein as a single point in such a 2D graph. Since extended disordered proteins typically contain fewer hydrophobic residues and more charged residues than ordered proteins, these two types occupy different areas in the CH-phase diagram and can even be separated by a linear boundary [29]. According to this analysis, all of the proteins located above this boundary line are highly likely to be disordered, whereas proteins below this line are structured. On the CH-plot, the vertical distance from the location of a protein to the boundary line is then taken as a scale of disorder (or structure) tendency of a protein. This distance is further referred as CH-distance.

CDF- and CH-plots have different underlying principles. The CDF-plot, being based on the disorder predictors of the PONDR^®^ family, is strongly related to the method of machine learning. Essentially, it is a statistical analysis based on known structures in PDB. The CH measurement has a very intuitive physicochemical background. Charged residues intend to interact with solvent molecules, while hydrophobic residues prefer to avoid contacts with solvent, therefore aggregating together. Hence, the CH-distance provides very important information about the general compactness and conformation of a polypeptide chain. By combining CDF- and CH-distances in one graph, we have another method called the CH-CDF-plot [37,52]. On this plot, each point corresponds to a single protein and represents its CDF-distance at the X-axis and the CH-distance at the Y-axis. CH>0 and CH<0 denote proteins predicted to be disordered and ordered by the CH-plot, respectively. On the other hand, values of CDF>0 represent structured proteins, and CDF<0 correspond to disordered proteins. Hence, the entire field can be divided into four quadrants by cutting lines CH=0 and CDF=0. Lower right quadrant corresponds to proteins predicted to be structured by both CH and CDF, whereas upper left quadrant contains proteins predicted to be is disordered by both methods.

### Composition profiling

To gain insight into the relationships between sequence and disorder, the amino acid compositions of Archaea proteomes were compared using an approach developed for the analysis of intrinsically disordered proteins [28,53]. To this end, the fractional difference in composition between a given protein set (an Archaean proteome), and proteins from the Fully Disordered Dataset (FDD) [46,54] was calculated for each amino acid residues as described in [28,53]. The fractional difference was calculated as (C_X_-C_FDD_)/C_FDD_, where C_X_ is the content of a given amino acid in a given proteome, and C_FDD_ is the corresponding content in FDD proteins. These fractional differences for each proteome are then plotted for each amino acid. This analysis was performed using a Composition Profiler, a computational tool that automates this task and graphically summarizes the results [53]. Composition Profiler is available at http://profiler.cs.ucr.edu.

## Results and discussion

### Major characteristics of the Archaea proteomes

Archaea are very abundant in nature, play a number of important roles in the cycle of carbon and nitrogen on earth [18]. Although most of archaea live in ocean, many of these microbes are extremophiles since they live, grow and prosper in extremely harsh environments, such environments of highly salty lakes or hot/boiling springs. For the cells of “normal” organisms (e.g., mammals), these types of environment are absolutely lethal, since high temperatures or high salt concentrations will inevitably denature proteins of these organisms, invalidate their functions, and terminate crucial biological pathways, eventually leading to the cell death. However, compared to these normal cells, archaea developed special mechanisms to counteract the harmful influence of these environments. The major components involved in these protective mechanisms should directly target the most abundant bio-substance: proteins. Therefore, the comparative analysis of proteomes of various species living at various habitats should provide crucial information on the similarities and differences of these organisms and on the mechanisms of the adaptation.

**Figure 2:**
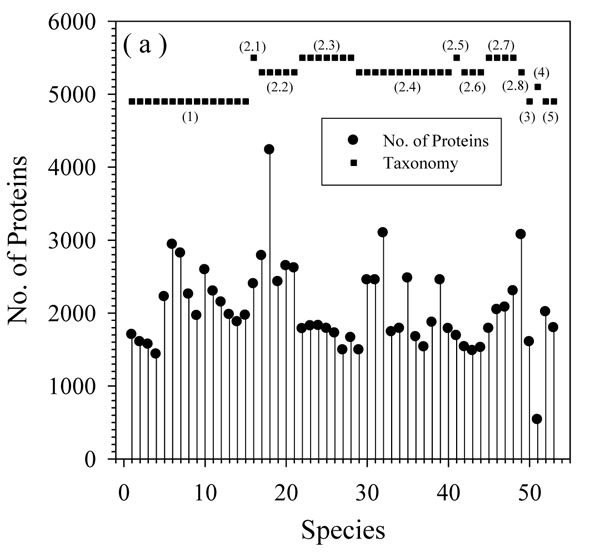
**Size of proteome of each species:** The X-axis is the index of the number of each species, while the Y-axis is the number of proteins. Filled circles represent the size of the proteome of each species. Filled squares indicate the taxonomy of archaea with similar species together and on same level. (1), (3)-(5) indicate species in four phyla: **Crenarchaeota**, **Korarchaeota**, **Nanoarcgaeota,** and **Thaumarchaeota**. (2.1) - (2.8) are eight different classes in the phylum of **Euryarchaeota** as shown in Table. 1.

Figure 2 presents the size distribution of proteomes of various archaea species analyzed in this study. Although 15 species in the first phylum, **Crenarchaeota**, belong to the same class, they can be divided into three orders: the first order is ***Desulfurococcales*** with 4 species; the second order is ***Sulfolobales*** having another 4 species; and the last order is ***Thermoproteales*** which contains 7 species. After this division is taken into account, the trends in the proteome sizes of these 15 species became obvious. Figure 2 shows that the members of the ***Desulfurococcales*** order are relatively uniform and have the smallest proteomes size in this phylum. Two other orders (***Sulfolobales*** and ***Thermoproteales***) still possess large variability in their proteome sizes. In **Euryarchaeota**, as shown by taxonomy (2.1) – (2.7) and corresponding proteome size in Figure 2, ***Halobacteria*** has the largest proteomes; ***Methanococci*** and ***Thermococci*** have fewer proteins in their proteomes; whereas ***Methanomicrobia*** have the largest fluctuations in proteome size among various species. Apparently, all the species with small proteomes are characterized by a globule-like morphology. The relatively large number of proteins in ***Halobacteria*** is also expected, since extra proteins may be needed to help these species deal with the high concentrations of ions in their environment. Finally, *Uncultured methanogenic archaeon* which belong to the **Euryarchaeota** phylum has more than 3000 proteins and ranks as one of the largest proteomes in Archaea. **Korarchaeota** and **Thaumarchaeota** have middle-sized proteomes. **Nanoarchaeota** have the only representative *Nanoarchaeum equitans*, which is the simplest species in Archaea being characterized by the smallest proteome and having only 536 proteins.

**Figure 3:**
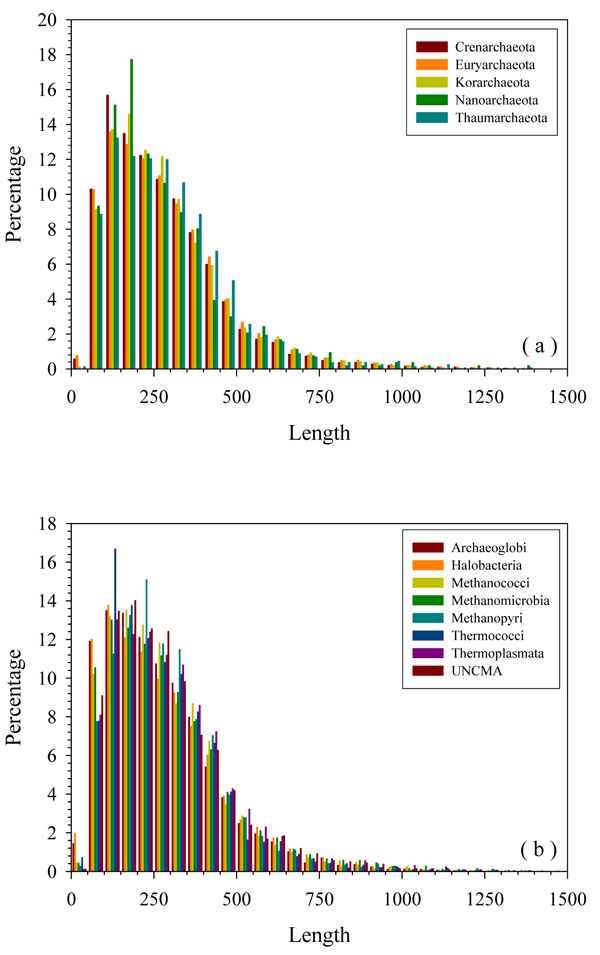
**Length distribution of proteins in five phyla (a) and eight classes (b) of Euryarchaeota**. X-axis: “X” length of protein; Y-axis: percentage of proteins with “X” length. The upper limit of the x-axis is taken as 1500 residues for visualization purposes. However, there are still scattered distributions of proteins beyond this uplimit.

Not only the size of proteome is important, but also the size of proteins in each genome. The length distributions for 5 phyla and 7 classes of **Euryarchaeota** phylum are shown in Figure 3(a) and Figure 3(b), respectively. Clearly, in general, distributions of protein length among all the species are very similar, although some important subtle differences can be found. The general shape of the distribution is similar to the power-law distribution. All of the species have less than 2% extremely short proteins (less than 50aa). The most optimal protein length for all species is around 100 – 200 residues. Proteins with these lengths constitute approximately 25% of any given proteome. Larger proteins are also very common in all the species: the content of proteins longer than 500aa is around 10% or even higher. Very long proteins (longer than 1000 aa) are not very common and account for several percent, comparable to the proportion of the extremely short proteins. As shown in Figure 3(a), **Thaumarchaeota** and **Korarchaeota** have fewer extremely short and short proteins. However, the members of the **Korarchaeota** phylum have more middle-sized proteins (150 – 250aa), whereas **Thaumarchaeota** have more proteins with 250 – 500 residues. In addition, **Nanoarchaeota** has around 5% percent more middle-sized proteins with 50 – 100 residues. In Figure 3(b), ***Archaeoglobi*** and ***Halobacteria*** have around 4 times more extremely short proteins than the other 5 classes. The other 5 classes are enriched in longer proteins with 250 – 450 residues. In ***Methanococci***, the content of proteins with 50 – 450 residues is always the highest.

### Amino acid compositions of the Archaea proteomes

**Figure 4:**
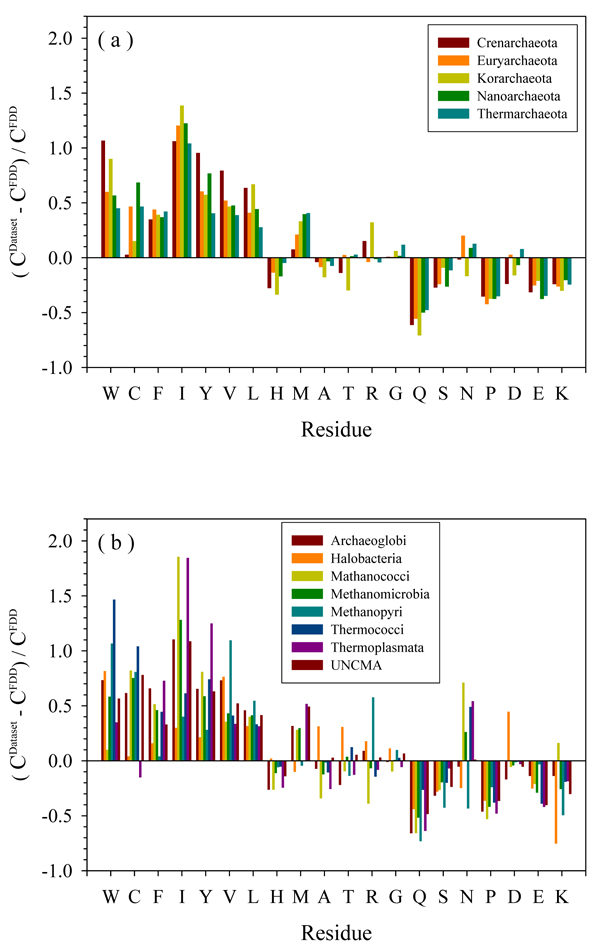
**Composition profile of amino acids for (a) five phyla, and (b) eight classes in Euryarchaeota:** Residues on the X-axis are arranged according to the increasing disorder tendency. Y-axis: the relative compositional profile compared to a fully disordered dataset.

At the next stage, the amino acid compositions of proteins from various Archaea were analyzed. The results of this analysis are shown in Figure 4 as the relative composition profiles calculated for various species as described by Vacic and colleagues [53]. Here, the fractional difference in composition between a given protein set and a set of completely disordered proteins was calculated for each amino acid residue. The fractional difference was evaluated as (C_X_-C_FDD_)/C_FDD_, where C_X_ is the content of a given amino acid in a given protein set, and C_FDD_ is the corresponding content in the Fully Disordered Dataset (FDD) [46,54]. The usefulness of this analysis is determined by the fact that the propensity of a given protein to be intrinsically disordered is determined by a set of specific features of its amino acid sequence and composition [28,29,50,53,55]. For example, intrinsically disordered proteins are significantly depleted in bulky hydrophobic (I, L, and V) and aromatic amino acid residues (W, Y, and F), which would normally form the hydrophobic core of a folded globular protein, and also possess a low content of C and N residues. These depleted residues, I, L, V, W, F, Y, C and N, were proposed to be called order-promoting amino acids. On the other hand, intrinsically disordered proteins and regions were shown to be substantially enriched in disorder-promoting amino acids: E, K, R, G, Q, S, P and A.

Application of this tool to the analysis of the Archaea proteomes revealed a number of interesting features. Figure 4(a) clearly shows that all 5 phyla contain many more structure-promoting residues (W, C, F, I, Y, V, L) and fewer disorder-promoting residues (Q, S, P, E, K) than the FDD proteins. In Figure 4(a), the content of F, I, Q, P, E, and K are rather consistent between the 5 phyla, where compared to FDD isoleucine had the highest abundance in all 5 phyla. By comparing each phylum, **Crenarchaeota** and **Korarchaeota** have more similarity with each other. They both have a low content of cystein, but are rich in tryptophan. In comparison with other phyla, they are also very similar in their content of H, T, R, and D. Figure 4(b) shows that the contents of L, Q, S, and P are stable among various classes of **Euryarchaeota**. ***Halobacteria*** are very special due to their low content of structure-promoting residues (C, F, I, and Y), abnormally large increments in A, T, R, G, and D, and a dramatic decrease in K abundance. ***Methanococci*** show a large increase in (I, N, and K) and a noticeable decrease in (H, A, and R). ***Methanopyri*** show a very large increase in V, and R, and a decrease in S, N, and K. ***Thermoplasmata*** have much more I, Y, and M than other classes.

Table 1 presents the averaged Kullback-Leibler [54,56,57] divergence among all the phyla and classes. As stated in our previous study, values below 0.01 correspond to high similarities between two datasets; values between 0.01 and 0.05 indicate a gray zone; values larger than 0.05 correspond to the datasets which are unlikely to be similar; and values greater than 0.1 correspond to the non-similar datasets [54]. This Table provides a very straightforward description of the similarity among the various Archaea species and shows that several species are similar to each other in terms of their amino acid compositions.

**Table 1:** Kullback-Leibler (KL) distances among 12 classes of 5 archaea phyla.

	(1)	(2.1)	(2.2)	(2.3)	(2.4)	(2.5)	(2.6)	(2.7)	(2.8)	(3)	(4)	(5)
(1)	1	0.012	0.091	0.065	0.019	0.049	0.012	0.041	0.016	0.015	0.076	0.027
(2.1)		1	0.107	0.046	0.017	0.051	0.008	0.042	0.022	0.016	0.068	0.026
(2.2)			1	0.214	0.088	0.059	0.135	0.158	0.068	0.110	0.271	0.074
(2.3)				1	0.034	0.170	0.040	0.028	0.062	0.070	0.022	0.052
(2.4)					1	0.078	0.023	0.022	0.007	0.026	0.065	0.005
(2.5)						1	0.071	0.147	0.065	0.055	0.200	0.079
(2.6)							1	0.035	0.032	0.018	0.044	0.035
(2.7)								1	0.036	0.041	0.048	0.030
(2.8)									1	0.029	0.094	0.006
(3)										1	0.089	0.032
(4)											1	0.091
(5)												1

### Disorder distribution in the Archaea proteomes

**Figure 5:**
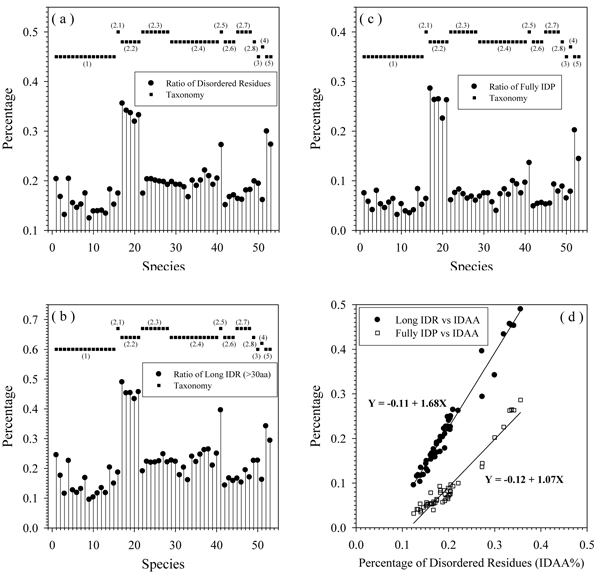
**Various measures of intrinsic disorder content in 53 species:** (a), Ratio of disordered residues in each species; (b), Percentage of proteins with long disordered regions (>30aa) in each species; (c), Ratio of fully disordered proteins in each species. In all figures above, the X-axis is the index number of each species. Filled squares indicate the taxonomy of archaea with similar species together and on the same level. (1), (3)-(5) indicate species in four phyla: **Crenarchaeota**, **Korarchaeota**, **Nanoarcgaeota**, and** Thaumarchaeota**. (2.1) - (2.8) are eight different classes in the phylum of **Euryarchaeota**. (d), Correlation among various disordered contents. X-axis: the ratio of disordered residues. Y-axis: percentage of proteins with long disordered regions and percentage of fully disordered proteins, accordingly.

The differences in the protein length distributions among the proteomes of various Archaea and in their amino acid compositions lead to another important question: Is intrinsic disorder distributed evenly in all these species or not? The comparison of various disorder contents among 53 species is shown in Figure 5, where the amount of disorder in different Archea proteomes is annotated as the percentage of predicted disordered residues (Figure 5(a)), the amount of long disordered regions (Figure 5(b)), and the amount of wholly disordered proteins (Figure 5(c)). Figure 5 clearly shows that **Crenarchaeota** have a relatively lower content of disorder than the other four phyla. On the other hand, **Korarchaeota** have the highest disorder content. In **Euryarchaeota**, the ratios diverged: ***Halobacteria*** have much higher disorder content than other species and ***Thermococci*** have the lowest disorder content. These observations indicated the influence of environment on the abundance of intrinsic disorder in a given organism. ***Halobacteria*** need to be more adaptive in signaling and regulation to counteract the high ion concentration. ***Thermococci*** tend to have more stable ordered proteins to resist the influence of high environment temperature.

Figure 5(d) represents the relation among the various means used to evaluate the disorder content in the Archaea proteomes. As shown by this plot, the total number of disordered residues, the amount of long IDRs, and the number of wholly disordered IDPs are well-correlated at the proteome level. In other words, this analysis clearly shows that the proteomes with the larger total amount of disordered residues typically contain a larger amount of long disordered regions and larger number of wholly disordered proteins.

**Figure 6:**
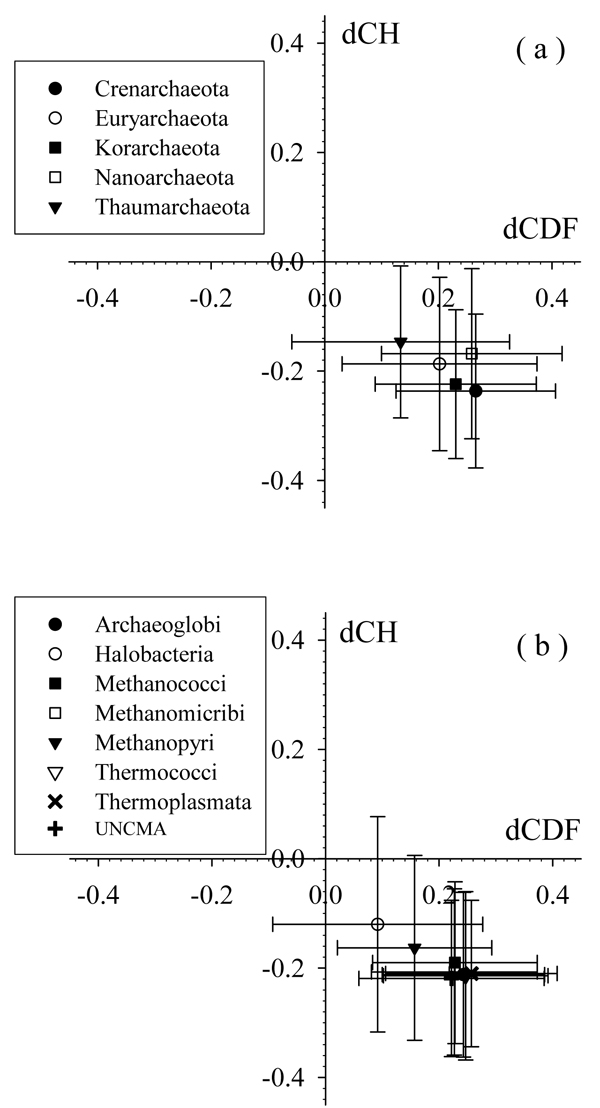
**Averaged CH-CDF plots for (a) five phyla and (b) eight classes in Euryarchaeota**. Various symbols indicate the averaged values of CH- and CDF-distances of all proteins in that species. Error bars are calculated from the root mean square deviation of the same set of proteins.

To better understand the distribution of wholly disordered proteins in various Archaea proteomes, we further analyzed their CH-CDF phase space. The averaged data for all 5 Archaea phyla and 8 classes of **Euryarchaeota** are shown in Figure 6(a) and Figure 6(b), respectively. As shown by Figure 6(a), the averaged CH-distance values are decreasing, while averaged CDF-distances are increasing in the order of **Thaumarchaeota**, **Euryarchaeota**, **Nanoarchaeota**, **Korarchaeota**, and **Crenarchaeota**. This trend indicates the correspondingly decreased content of charged residues, increased content of structured-promoting residues, or a combination of these two factors. Error bars give the estimation of the distribution of all the relevant distances for that species. Apparently, larger error bars correspond to a broader distribution. Hence, while the distributions of CDF-distances are similar among all five phyla, **Thaumarchaeota** has broadest distribution of the CH-distances. In Figure 6(b), ***Halobacteria*** and ***Methanopyri*** have obviously larger averaged CH-distance and smaller averaged CDF-distance than other 5 classes. ***Halobacteria*** has much broader distribution of the CDF-distances. The other 5 **Thaumarchaeota** classes have somewhat overlapped values.

The abundance of intrinsically disordered proteins in various Archaea proteomes is further illustrated by Figures S1 and S2 (see additional file 1) which represent CH-CDF plots for Archaea phyla (Additional file 1, Figure S1) and for the 8 **Euryarchaeota** classes (Additional file 1, Figure S2). In these plots, each spot corresponds to a single protein and its coordinates are calculated as a distance of this protein from the boundary in the corresponding CH-plot (Y-coordinate) and an averaged distance of the corresponding CDF curve from the boundary (X-coordinate). Positive and negative Y values correspond to proteins which, according to CH-plot analysis, are predicted to be natively unfolded or compact, respectively. Whereas positive and negative X values are attributed to proteins that, by the CDF analysis, are predicted to be ordered or intrinsically disordered, respectively. Therefore, each plot contains four quadrants: (-, -) contains proteins predicted to be disordered by CDF, but compact by CH-plot (i.e., proteins with molten globule-like properties); (-, +) includes proteins predicted to be disordered by both methods (i.e., random coils and pre-molten globules); (+, -) contains ordered proteins; (+, +) includes proteins predicted to be disordered by CH-plot, but ordered by the CDF analysis. Both figures also give the number of proteins found in the corresponding quadrants. Analysis of the (-, -) and (-, +) quadrants in Additional file 1, Figure S1 shows that the majority of the wholly disordered proteins from **Crenarchaeota**, **Korarchaeota**, **Euryarchaeota**, and **Thaumarchaeota** likely possess molten globule-like properties. In contrast, the proteomes of **Nanoarchaeota** are generally characterized by a more balanced distribution between compact and extended disordered proteins. The analysis of these two quadrants in the **Euryarchaeota** phylum (see Figure S2 in Additional file 1) shows that proteomes of ***Archaeoglobi***, ***Methanococci***, ***Methanomicrobia***, ***Methanopyri***, ***Thermococci***, and UNCMA all have more molten globule-like IDPs than extended IDPs. The situation is reversed in ***Halobacteria*** and ***Thermoplasmata*** which are predicted to have more extended IDPs than native molten globules.

### Intrinsic disorder and habitats of the Archaea

**Figure 7:**
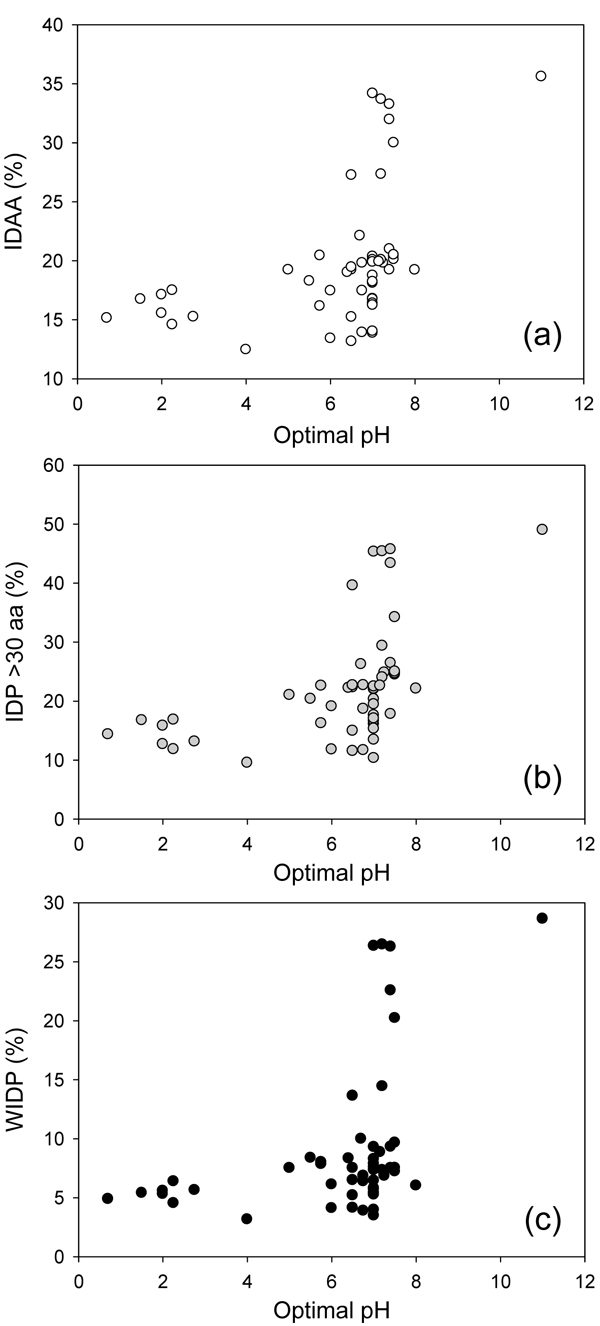
**The distribution of intrinsic disorder content in the Archaea as a function of optimal pH.** Three measures of intrinsic disorder: (a) overall percentage of intrinsically disordered residues (IDAA); (a) percentage of proteins with long disordered regions (IDP >30 aa); (c) and the percentage of wholly disordered proteins (WIDP).

In order to understand a correlation between the abundance of IDPs in various Archaea and their natural habitats, we searched for several environmental characteristics, such as optimal salinity, optimal pH and optimal temperature (see Table S1 in Additional file 1). Figure 7 represents the disorder content in the Archaea as a function of optimal pH. We used three measures of intrinsic disorder, overall percentage of intrinsically disordered residues (IDAA), percentage of proteins with long disordered regions (IDP >30 aa), and percentage of wholly disordered proteins (WIDP). Figure 7 shows that the organisms living in habitats with pH values close to neutral (ranging from pH 6.0 to pH 8.0) possess very large disorder diversity. On the other hand, all the acidophilic Archaea are characterized by the relatively low abundance of intrinsic disorder, whereas the only alkaliphile, *Natronomonas pharaonis*, has the highest content of intrinsic disorder as measured by the overall number of disordered residues, the number of long disordered regions and the number of completely disordered proteins in its proteome.

**Figure 8:**
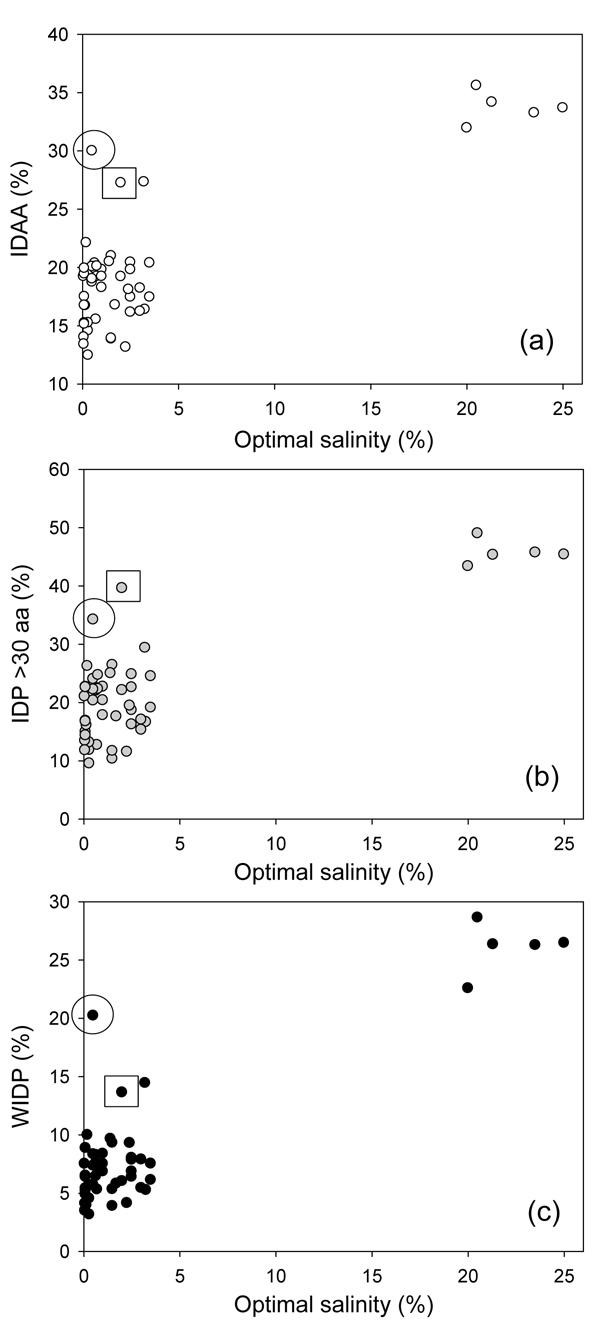
**The distribution of intrinsic disorder content in the Archaea as a function of salinity of their habitats.** Three measures of intrinsic disorder: (a) overall percentage of intrinsically disordered residues (IDAA); (b) percentage of proteins with long disordered regions (IDP >30 aa); (c) and the percentage of wholly disordered proteins (WIDP).

The dependence of the disorder content in the Archaea on the salinity of their habitats is shown in Figure 8, which clearly shows that all the halophiles are characterized by a very large amount of disorder. This observation supports the notion that extra IDPs are likely to be needed to these species to help them dealing with the high concentrations of ions in their environment. Of special interest is a *Cenarchaeum symbiosum*, which live in the low salinity environments but is still characterized by high abundance of IDPs (see circled point in Figure 8). The peculiar difference of this organism is that this symbiotic archaeon is a psychrophilic crenarchaeon which inhabits a marine sponge. Another peculiar organism with large amount of disorder is *Methanopyrus kandleri* (see squared point in Figure 8). Although the living environment of this archaeon is characterized by the normal salinity, it is known to grow at the hostile conditions of very high temperatures (between 100 and 110°C) and high hydrostatic pressure. In fact, *Methanopyrus kandleri* was isolated from the overheated walls of the black smoker from the Gulf of California found at the depth of 2000 m.

**Figure 9:**
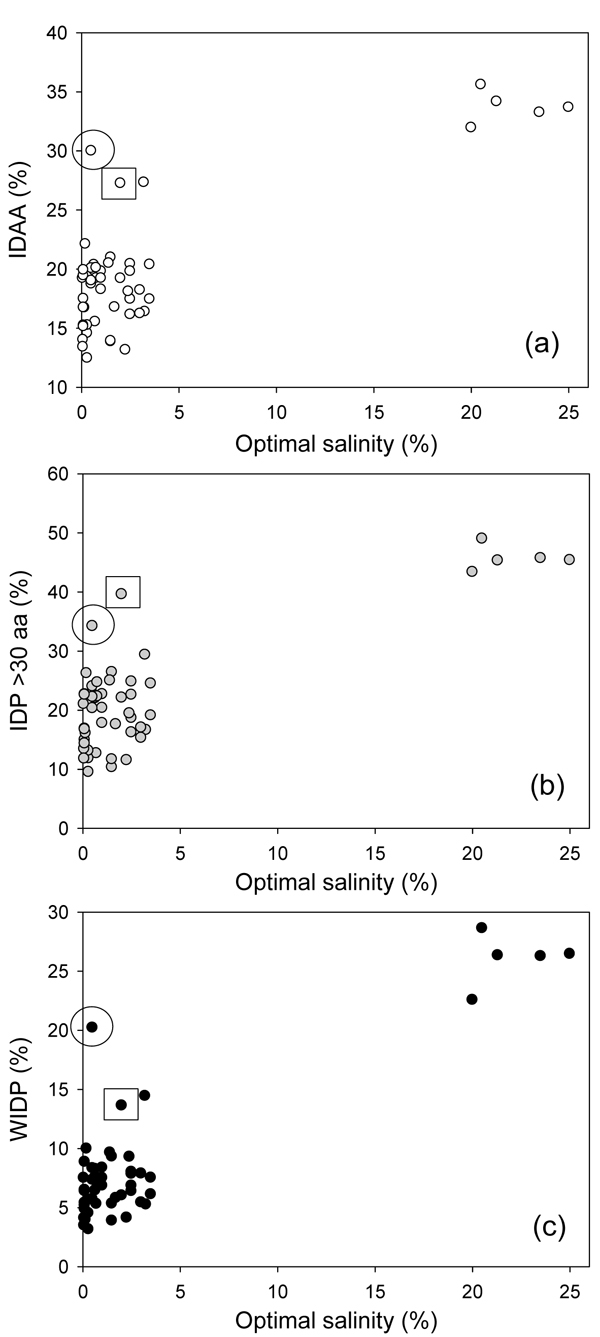
**The distribution of intrinsic disorder content in the Archaea as a function of temperature of their habitats.** Three measures of intrinsic disorder: (a) overall percentage of intrinsically disordered residues (IDAA); (b) percentage of proteins with long disordered regions (IDP >30 aa); (c) and the percentage of wholly disordered proteins (WIDP).

Finally, Figure 9 represents the dependence of the amount of disorder in various Archaea as a function of temperature of their habitats. Figure 9 shows that generally there is a slight negative correlation between these two parameters. The obvious exceptions from this trend are halophilic proteomes (see squared points in Figure 9), as well as already discussed *Methanopyrus kandler* (see triangled point in Figure 9) and *Cenarchaeum symbiosum* (see circled point in Figure 9).

Altogether, data represented in Figure 7, 8 and 9 show that the amount of intrinsic disorder in Archaea correlates with the peculiarities of their environment. Generally, organisms prospering at the extremely hazardous conditions (such as very high temperature, highly alkaline pH, very high salinity) are enriched in IDPs. Of special interest is the fact that various environmental factors possess different strength in promoting intrinsic disorder. For example, organisms living in an extremely hostile, highly acidic environment possess relatively low amount of disorder. Even proteins of the archaeon *Picrophilus torridus* which lives in and grows at the lowest pH values known among all organisms, including conditions such as 1 M sulfuric acid, effectively grows only below pH 3.5 (optimal pH = 0.7) and possesses significant growth even at a pH around 0, contain only 15% of disordered residues. On the other hand, among the most prominent enhancers of intrinsic disorder are habitats with very high salinity and alkaline pH. The combination of extremely high temperature and high hydrostatic pressure potentially also represent environment favoring intrinsic disorder. Another strong disorder-promoting factor is the symbiotic life style. All this suggests that intrinsic disorder can be used by the Archaean organisms to better adjust for their harsh living conditions or, in the case the symbiotic microbes, for the accommodation to the conditions inside the sponge and for better communication with the cells of the host.

### Intrinsic disorder and function of the Archaea proteins

Earlier studies clearly showed that protein intrinsic disorder is of great functional importance [27,31,32]. Proteins often contain one or more functional *domains*, different combinations of which give rise to the diverse range of proteins found in nature. It has been recognized that the identification of domains that occur within proteins can therefore provide insights into their function. To find a correlation between intrinsic disorder and function in the Archaea proteins we analyzed the abundance of intrinsic disorder in the Pfam database, which contains information on protein domains and families and uses hidden Markov models and multiple sequence alignments to identify members of its families emphasizing the evolutionary conservation of protein domains [58-60]. Each curated family in Pfam is represented by a seed and full alignment. The seed contains representative members of the family, while the full alignment contains all members of the family as detected with a profile hidden Markov model (HMM) [58]. Since Pfam represent an important tool for understanding protein structure and function and since this database contains large amount of information on functional domains, the Archaea seed domains in the version 23.0 of the Pfam database were analyzed. There are more than 12,700 Pfam domain seeds of the Archaean origin, which vary in length from 16 to 1462 residues, whereas the mean length of the Archaean Pfam domains is 156 residues (Figure S3, in Additional file 1).

Figure S4 in Additional file 1 ) shows that intrinsic disorder is rather abundant in the Archaean Pfam seed domains. On average, 15.4 % of residues in functional domains of the Archaea origin are predicted to be disordered (Figure S4A). In fact, several Archaean domains are completely disordered and only ~2,000 domains are completely devoid disordered regions (Figure S4A, additional file 1). Many of the domains contain at least one disordered region, with some domains possessing more than 10 disordered regions (Figure S4B, Additional file 1). The length of disordered regions in the domains varies from 1 to 201 residues (Figure S4C, Additional file 1).

The intrinsic disorder propensity among the Archaean members of the Pfam database is further illustrated by Figure S5 (see Additional file 1) which represents a three dimensional plot of total percent disorder, disordered region length (where there are up to 26 disordered regions per domain), and domain length for all Archaea seed domains in version 23.0 of the Pfam database. Figure S6 (see Additional file 1) represents the data as a three dimensional plot of the log of the number of disordered regions, the log of the number of disordered residues, and the log of the percent disorder in each of all of the Archaea seed domains in version 23.0 of the Pfam database. In this plot, all domains with one disordered region are represented in the cluster on the left. Domains with between ten and 26 disordered regions are represented at 1 and above on the right. Domains with no disordered regions are not included. These figures suggest that there is a weak correlation between percent disorder and disordered region length, but no correlation between these observations and Pfam domain length.

Therefore, data presented here clearly show that many functional domains in Archaea are predicted to contain various amounts of disordered residues. Table 2 lists several domains with high disorder content and shows that these intrinsically disordered domains play crucial roles in interaction with RNA, DNA, and proteins and are important for recognition, regulation and signal transduction. In other words, Archaean disordered domains fulfill functions similar to those of prokaryotic and eukaryotic proteins [27,31,32].

**Table 2:** Illustrative highly disordered Pfam domains of archaean origin

Name	Domain	Description	% D
NOP10_SULSO	1-53	nucleolar protein essential for normal 18S rRNA production and rRNA pseudouridylation	41
PUR2_METJA	1-102	related to the N-terminal domain of biotin carboxylase/carbamoyl phosphate synthetase	42
O27142_METTH	17-302	tldD and pmbA proteins, found to suppress mutations in letD and inhibit of DNA gyrase	44
Y2068_ARCFU	10-100	transmembrane region of Cytochrome C biogenesis protein believed to bind double-stranded DNA	64
RF1_METTH	2-137	eRF1 stops protein biosynthesis by recognising stop codons and stimulating peptidyl-tRNA hydrolysis	81
			
Y2677_METMA	7-59	CsbD, a bacterial general stress response protein	100
MTPE_SULTO	1-56	epsilon subunit of the ATP synthase, a potent inhibitor of ATPase activity	100
Q48297_HALSA	295-353	helical bundle domain, homodimer interface of the signal transducing histidine kinase family	100
Q8TTT9_METAC	235-302	NosD, a periplasmic protein thought to insert copper into the exported reductase apoenzyme	100

**Figure 10:**
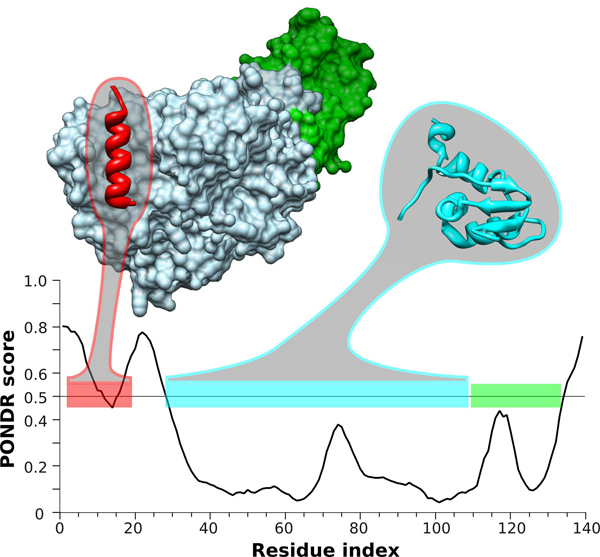
**PONDR^®^ prediction and experimentally solved structure of aIF2β from *Sulfolobus solfataricus*.** The PONDR^®^ VSL2 prediction is given in the plot, where scores greater than 0.5 are predictions of disordered residues and scores less then 0.5 are predictions of ordered residues. Horizontal bars represent regions with known structure, or are likely to be structured, which are (from N- to C- termini): the aIF2γ-binding MoRF region (red bar), the core domain (cyan bar), and the C-terminal zinc finger domain (green bar). Additionally, structures of the aIF2γ-binding MoRF region (red ribbon) bound to aIF2γ (blue surface) and aIF2α (green surface), and of the core domain (cyan ribbon) are shown (coordinates from PDB entries 2QN6 and 2NXU, respectively).

Further evidence on the biological importance of intrinsic disorder found in the archaea proteins is given by Figure 10 which illustrates predicted and experimentally verified disordered regions in the Archaea translation initiation factor 2 (aIF2). aIF2 facilitates translation by recruiting methonyl-tRNA to the ribosome and aiding in the identification of the start codon, hydrolyzing GTP in the process [61]. aIF2 consists of three subunits: regulatory α and β subunits, and the GTP hydrolyzing γ subunit. aIF2β of *Sulfolobus solfataricus* is an intrinsically disordered protein consisting of both ordered and disordered regions (Figure S5, see additional file 1). The N-terminus of aIF2β has been shown to be disordered [62] and also in the homologous protein from *Methanobacterium thermoautotrophicum*[63]. However, this region is responsible for mediating binding to aIF2γ through a MoRF-type interaction. This interaction is shown in Figure 10, where the aIF2γ binding region corresponds to a local prediction of order in the N-terminus of aIF2β. Additionally, aIF2β has a central core domain and a C-terminal zinc finger domain, both of which play roles in RNA recognition [64]. Presumably the MoRF interaction provides flexibility to these domains to facilitate molecular recognition [64].

### Phylogenetic tree of the Archaea and intrinsic disorder

**Figure 11:**
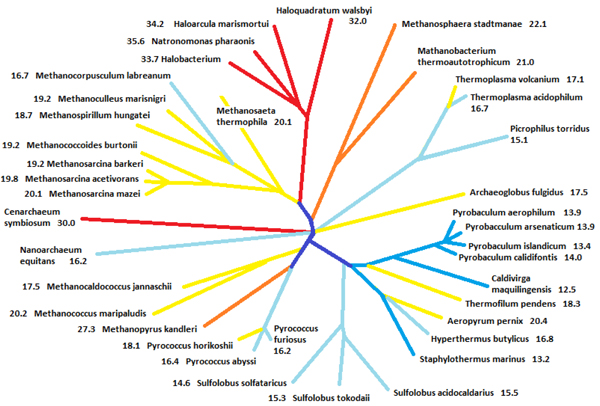
**Intrinsic disorder and phylogenetic tree of Archaea.** This schematic figure is made manually by taking the figure in http://archaea.ucsc.edu as a template. Several species not analyzed in our study were removed. Distances between very similar species were also intentionally increased. Numbers nearby the species name represent the relative content of disordered residues in that species. Colors of the tree are assigned according to the abundance of disordered residues (red:>30%; orange: >21%; yellow: >17%; light blue: >14%; dark blue:<=14%).

Figure 11 overlaps the disorder content of various species with the Archaea phylogenetic tree. In this figure, colors of the branches correspond to the abundance of disordered residues in the corresponding species. Clearly, as indicated by the same color on the related branches of the tree, proteomes belonging to the same phylum typically have comparable contents of disordered residues in their proteins. For example, all the species in the **Crenarchaeota** phylum are characterized by the relatively small amount of disordered proteins, containing in average ~15% intrinsically disordered amino acids (IDAA). The correlation is even stronger for species belonging to the same order, where the amount of intrinsic disorder remains relatively constant. For example, all the species from the ***Thermoproteales*** order have low IDAA content (less than 14%). This value increases to ~16% in various ***Sulfolobales*** and further increases to up to 20% in ***Desulfurococcales***.

Analysis of the **Eutyarchaeota** phylum also revealed a comparable trend in the distribution of IDAA. Here, all the members of the ***Archaeoglobi***, ***Halobacteria***, ***Methanococci***, and ***Methanomicrobia***, contain a relatively high amount of disorder (ranging from ~16 to ~36%). Once again, each **Eutyarchaeota** class was characterized by the relatively uniform distribution of disorder: for example, the amount of disorder in the ***Halobacteria*** ranged from 32 to 35.6%, whereas ***Methanomicrobia*** contained from 16.7 to 20.1 % IDAA.

Interestingly, Figure 11 provides some insights into the correlation between the evolution of Archaea and the intrinsic disorder distribution in these organisms. In the ***Methanococci*** – ***Methanopyri*** – ***Thermococci*** branch, ***Methanoccoci*** deviated first from other Archaea with the high IDAA of ~20%. Later on, ***Methanopyri*** left the main branch with ratio of IDAA up to 27%. Although ***Thermococci*** generally possess a relatively low amount of disorder in comparison with other members of this branch, *Pyrococcus horikoshi* being close to ***Methanoccoci*** and ***Methanopyri***, is characterized by the highest disorder content (~18%), whereas other members of this class are close to the **Crenarchaeota** phylum and are correspondingly characterized by the lower amount of disorder (~16%). In the **Crenarchaeota** phylum, where the majority of members are characterized by the disorder content ranging from 12.5 to 14.0%, *Hyperthermus butylicus*, *Thermofilum pendens*, and *Aeropyrum pernix*, all located in the close branches, which deviated from the major branch relatively late, possess 17-20% IDAA. Therefore, these observations suggest that in general the amount of disorder increases with evolution. There is only one counter-example to this rule, which is found in the class of ***Methanoicrobia***, where *Methanocorpusculum labreanum* is not the oldest species in that class, but has an apparently lower content of disordered residues than other older species.

### Need for the habitat-specific disorder predictors

Data presented in this paper indicate that there is a correlation between the amount of intrinsic disorder in a proteome of a given archaeaon and the peculiarities of its habitat. Intriguingly, not only the amounts of intrinsic disorder in the proteomes of archaea prospering in various hostile conditions are different and depend on the environmental peculiarities, proteins of these proteomes possess a number of environment-dependent characteristic features (e.g., specific biases in the amino acid compositions). Data shown in Figure 4(b) suggest that these sequence features are unique and different enough to potentially allow the development of habitat-specific predictors of intrinsic disorder for archaea. In fact, this hypothesis is in agreement with our recent study of integral transmembrane proteins which revealed that the disordered regions from helical bundle integral membrane proteins, those from β-barrel integral membrane proteins, and those from water soluble proteins all exhibit statistically distinct amino acid compositional biases [54]. Although the detailed analysis showed that, despite these differences in composition, current algorithms make reasonably accurate predictions of disorder for these membrane proteins, it has been proposed that developing new predictors that make use of data from disordered regions in helical bundles and beta barrels will likely lead to significantly more accurate disorder predictions for these two classes of integral membrane proteins [54].

## Conclusions

In this paper, we systematically analyzed the abundance of intrinsically disordered proteins and the intrinsically disordered regions in 53 Archaea species, which are grouped into 5 phyla and 11 classes. The size of proteomes of these species extends from 536 proteins to 4,234 proteins with the majority of Archaea having around 2,000 proteins. The abundance of intrinsic disorder was species-dependent. The averaged ratio of predicted disordered residues varied from ~14% in ***Thermoproteales*** to ~34% in ***Halobacteria***. Further analysis based on amino acid composition profiles confirmed large differences between various species. However, even between closely related species, the content of disordered residues changed greatly. *Staphylothermus marinus* and *Ignicoccus hospitalis* are two species in the same order ***Desulfurococcales*** of the ***Thermoprotei*** class in the **Crenarchaeota** phylum, but *Ignicoccus hospitalis* had 7% more disordered residues than *Staphylothermus marinus*. In ***Thermoproteales*** of the same phylum and class, *Thermofilum pendens* had around 6% more disordered residues than *Caldivirga maquilingensis*.

The relation between various measures of disordered content; i.e., the relative content of disordered residues, the content of proteins containing long disordered regions, and the number of fully disordered proteins was also analyzed. All of these measures of intrinsic disorder content are shown to be linearly correlated with each other at the genome level. This relationship provided important information for the general understanding of disordered proteins. However, more computational experiments are needed to verify this conclusion since this result comes from the predictions on 53 species.

Next we analyzed the correlation between the abundance of intrinsic disorder in a given Archaeaon and peculiarities of its habitat. Since many of the Archaea are know to survive at extremely harsh environmental conditions, this exercise was interesting and important. Analysis revealed that various environmental factors possessed different strength in promoting intrinsic disorder. The most prominent enhancers of intrinsic disorder were habitats with very high salinity, alkaline pH or characterized by the combination of extremely high temperature and high hydrostatic pressure. Symbiotic archaeaon, *Cenarchaeum symbiosum*, was also shown to contain high level of intrinsically disordered proteins. This clearly suggested that Archaea generally utilized intrinsic disorder for adjustment to their living conditions.

Many functional Pfam seed domains of the Archaea origin were shown to possess various levels of intrinsic disorder. Only about 15% of these functional domains were completely devoid of disorder. Disordered Pfam domains were involved in various crucial functions, such as signaling, regulation and interaction with nucleic acids and proteins, suggesting that similar to proteins from other domains of life, intrinsic disorder is heavily used by the Archaean proteins in their functions.

We also designed a new protocol by combining disorder predictions and phylogenetic tree to show the correlation between evolutionary development and disorder. A gradual increase in the amount of intrinsic disorder with the evolution of species was observed. More interestingly, the ratios of disordered residues can also be reduced in the process of evolution. Based on the hypothesis that disordered proteins are crucial for signaling and regulation, it is not difficult to understand the need for an increased level of intrinsic disorder in newly evolved species. However, data for *Methanocorpusculum labreanum* raised the question on whether the decreased amount of intrinsic disorder found in this organism can be considered as an atavism. In fact, one of the *Methanocorpusculum labreanum* paralogues, *Methanosaete thermophila*, has a smaller proteome but higher content of disordered residues, whereas two other paralogues, *Methanoculleus marisnigri* and *Methanospirillum hungatai*, have a higher content of disordered residues and larger proteomes.

## Competing interests

The authors declare that they have no competing interests.

## Authors' contributions

BX, RWW, and CJO designed and implemented experiments. All authors analyzed results. VNU developed strategy and provided advice. Each author contributed equally in writing the paper. All authors read and approved the final manuscript.

## Supplementary Material

Additional file 1 Click here for file
