# Supplementary material for: Archaic chaos: intrinsically disordered proteins in Archaea
Source: BMC Syst Biol. 2010 May 28;4(Suppl 1):S1. doi: 10.1186/1752-0509-4-S1-S1 (PMC2880407; doi:10.1186/1752-0509-4-S1-S1)
Supplement: Additional file 1 [file 1752-0509-4-S1-S1-S1.Pdf]

Additional materials for

Archaic Chaos:
Intrinsically Disordered Proteins in Archaea

Bin Xue[1,2], Robert W. Williams[3], Christopher J. Oldfield[2,4], A. Keith Dunker[1,2] and Vladimir N. Uversky[1,2,5,*]

[1]Center for Computational Biology and Bioinformatics, Indiana University School of Medicine, Indianapolis, IN 46202, USA;

[2]Institute for Intrinsically Disordered Protein Research, Indiana University School of Medicine, Indianapolis, IN 46202, USA;

[3]Department of Biomedical Informatics, Uniformed Services University, Bethesda, MD 20814

[4]Center for Computational Biology and Bioinformatics, Indiana University School of Informatics, Indianapolis, IN 46202, USA;

[5]Institute for Biological Instrumentation, Russian Academy of Sciences, 142290 Pushchino, Moscow Region, Russia;

Table S1. List of all the archaea analyzed in this study, the number of proteins in their genomes, and some peculiarities of their habitats

Phylum	Class	Order	species	ExPASy ID	No. of proteins	$T_{optimum}$	$pH_{optimum}$	$Salinity_{optimum}$ (%)	Reference
Crenarchaeota (1)	Thermoprotei	Desulfurococcales	Aeropyrum pernix	AERPE	1702	90-95	7.5	3.5	[60]
			Hyperthermus butylicus (strain DSM 5456 / JCM 9403)	HYPBU	1602	95-106	7.0	1.7	[61]
			Staphylothermus marinus (strain ATCC 43588 / DSM 3639 / F1)	STAMF	1570	98	6.5	1-3.5	[62]
			Ignicoccus hospitalis (strain KIN4/I / DSM 18386 / JCM 14125)	IGNH4	1434	78-98	5-6.5	1-4	[63]
		Sulfolobales	Sulfolobus acidocaldarius	SULAC	2221	75-80	1-3	0.7	[64]
			Sulfolobus solfataricus	SULSO	2938	80	2-2.5	0.3	[65]
			Sulfolobus tokodaii	SULTO	2817	80	2.5-3	0.3	[66]
			Metallosphaera sedula (strain ATCC 51363 / DSM 5348)	METS5	2256	70	2-2.5	0.1	[67]
		Thermoproteales	Caldivirga maquilingensis (strain DSMZ 13496 / IC-167)	CALMQ	1962	85	3.7-4.2	0.3	[68]
			Pyrobaculum aerophilum	PYRAE	2590	100	7.0	1.5	[69]
			Pyrobaculum arsenaticum (strain DSM 13514 / JCM 11321)	PYRAR	2296	95	6.5-7.0	0-3	[70]
			Pyrobaculum calidifontis (strain JCM 11548 / VA1)	PYRCJ	2148	90-95	7.0	0.07	[71]
			Pyrobaculum islandicum (strain DSM 4184 / JCM 9189)	PYRIL	1976	100	5-7	0.07	[72]
			Thermofilum pendens (strain Hrk 5)	THEPD	1876	85-90	5.5	0.1-2	[73]
			Thermoproteus neutrophilus (strain DSM 2338 / JCM 9278 / V24Sta)	THENV	1965	85	6.5	0.1	[73]
Euryarchaeota	Archaeoglobi		Archaeoglobus fulgidus	ARCFU	2398	83	5.5-8.0	0.5-4.5	[74, 75]

(2)	(2.1)								
	Halobacteria (2.2)		Natronomonas pharaonis (strain DSM 2160 / ATCC 35678)	NATPD	2784	43-45	11	20.5	[76]
			Haloarcula marismortui	HALMA	4234	40-50	7.0	19.8-22.8	[77]
			Halobacterium salinarium	HALSA	2426	49-50	7.2	25	[78]
			Haloquadratum walsbyi (strain DSM 16790)	HALWD	2645	45	7.4	20	[79]
			Halobacterium salinarum (strain ATCC 29341 / DSM 671 / R1)	HALS3	2615	50	7.4	23.5	[80]
	Methanococci (2.3)		Methanocaldococcus jannaschii	METJA	1782	85	6.0	3.5	[81]
			Methanococcus maripaludis (strain C5 / ATCC BAA-1333)	METM5	1821	38	6.8-7.2	0.64	[82]
			Methanococcus maripaludis (strain C6 / ATCC BAA-1332)	METM6	1825	38	6.8-7.2	0.64	[82]
			Methanococcus maripaludis (strain C7 / ATCC BAA-1331)	METM7	1786	38	6.8-7.2	0.64	[82]
			Methanococcus maripaludis	METMP	1722	38	6.8-7.2	0.64	[82]
			Methanococcus aeolicus (strain Nankai-3 / ATCC BAA-1280)	META3	1490	46	6.5-8.0	0.5-4.5	[83]
			Methanococcus vannielii (strain SB / ATCC 35089 / DSM 1224)	METVS	1661	37-42	8.0	2.0	[84]
	Methanomicrobia (2.4)		Methanosarcina acetivorans	METAC	1490	35-40	6.5-7.0	1.0	[85]
			Methanosarcina barkeri (strain Fusaro / DSM 804)	METBF	2450	37-42	6.0-7.0	0.5-1.0	[86]
			Methanococcoides burtonii (strain DSM 6242)	METBU	2450	1-2	7.4	1.0	[87]
			Methanospirillum hungatei (strain JF-1 / DSM 864)	METHJ	3095	30-37	6.6-7.4	0.5	[73]
			Methanocorpusculum labreanum (strain ATCC 43576 / DSM 4855 / Z)	METLZ	1739	37	7.0	0.0-0.3	[88]
			Methanosarcina mazei	METMA	1786	40	7.2	0.5	[89]

			Methanoculleus marisnigri (strain ATCC 35101 / DSM 1498 / JR1)	METMJ	2476	20-25	6.4	0.5	[90]
			Methanosaeta thermophila (strain DSM 6194 / PT)	METTP	1673	55-60	7.5	0.5-1.0	[91]
			Methanosphaera stadtmanae (strain DSM 3091)	METST	1533	36-40	6.5-6.9	0.2	[92]
			Methanobacterium thermoautotrophicum	METTH	1869	65-70	7.2-7.6	1.5	[93]
			Methanoregula boonei (strain 6A8)	METB6	2450	37	5.0	0.05	[94]
			Methanobrevibacter smithii (strain PS / ATCC 35061 / DSM 861)	METS3	1783	37	7.5	0.08	[95]
	Methanopyri (2.5)		Methanopyrus kandleri	METKA	1687	100-110	6.5	2.0	[96]
	Thermoplasmata (2.6)		Picrophilus torridus	PICTO	1535	60	0.7	0.1	[97]
			Thermoplasma acidophilum	THEAC	1482	59	1.0-2.0	0.1	[98]
			Thermoplasma volcanium	THEVO	1523	60	2.0		[99]
	Thermococci (2.7)		Pyrococcus abyssi	PYRAB	1786	96	7.0	2.5-4.0	[100]
			Pyrococcus furiosus	PYRFU	2045	100	7.0	1.0-5.0	[101]
			Pyrococcus horikoshii	PYRHO	2077	98	7.0	2.4	[102]
			Pyrococcus kodakaraensis	PYRKO	2301	95	7.0	3.0	[103]
	Environment Samples (2.8)		Uncultured methanogenic archaeon RC-I	UNCMA	3071	35-37	6.5-7.8	0.1	[104]
Korarchaeota (3)			Korarchaeum cryptofilum (strain OPF8)	KORCO	1602	85	6.5	0.1	[106]
Nanoarchaeota (4)			Nanoarchaeum equitans	NANEQ	536	90	5-6.5	1-4	[15]
Thaumarchaeota (5)			Cenarchaeum symbiosum	CENSY	2014	8-18	7.5	0.5	[25]
			Nitrosopumilus maritimus (strain SCM1)	NITMS	1795	20-21	7.2	3.2	[105]

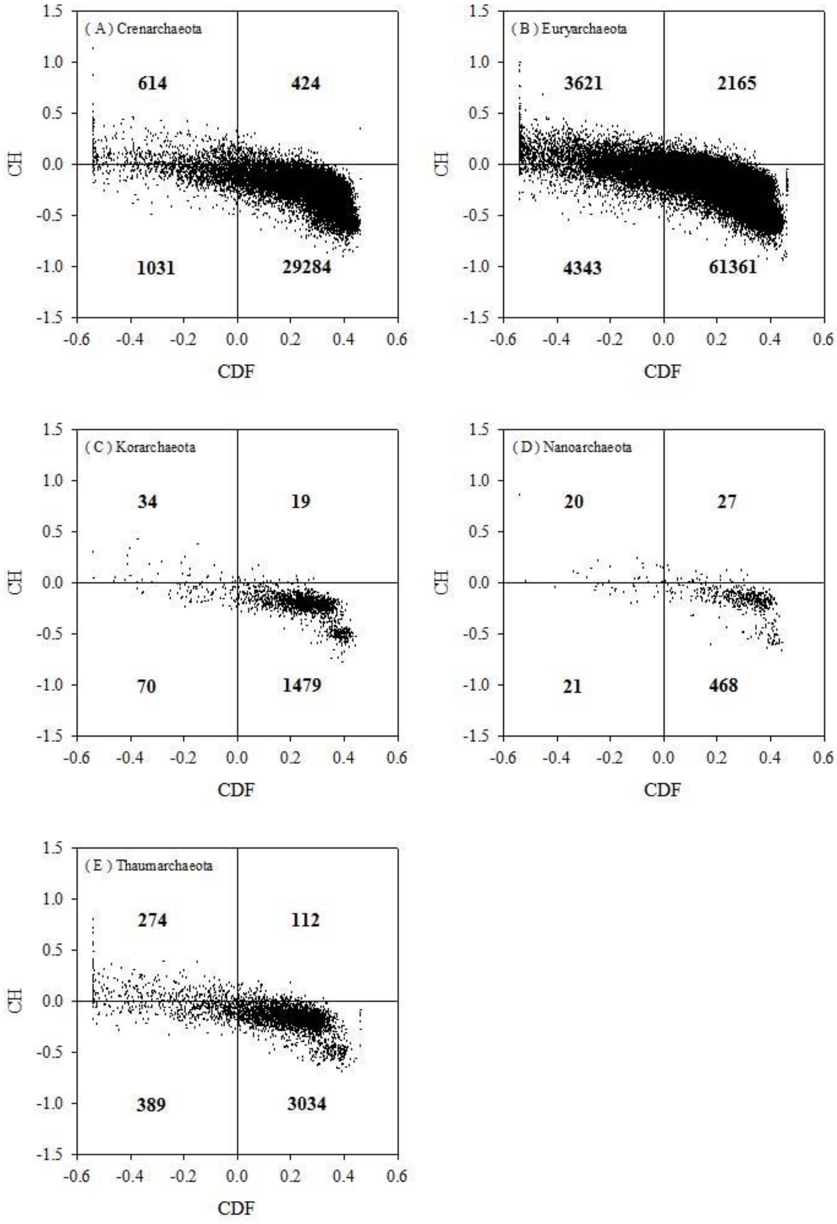

Figure S1. Comparison of the PONDR® VSL2 CDF and CH-plot analyses of whole protein order-disorder via distributions of proteins in each Archaea phyla within the CH-CDF phase space. Each spot represents a single protein whose coordinates were calculated as a distance of this protein from the boundary in the corresponding CH-plot (Y-coordinate) and an averaged distance of the corresponding CDF curve from the boundary (X-coordinate). Four quadrants in each plot correspond to the following predictions: (-, -) proteins predicted to be disordered by CDF, but compact by CH-plot; (-, +) proteins predicted to be disordered by both methods; (+, -) contains ordered proteins; (+, +) includes proteins predicted to be disordered by CH-plot, but ordered by the CDF analysis. Numbers correspond to numbers of proteins found in the corresponding quadrants.

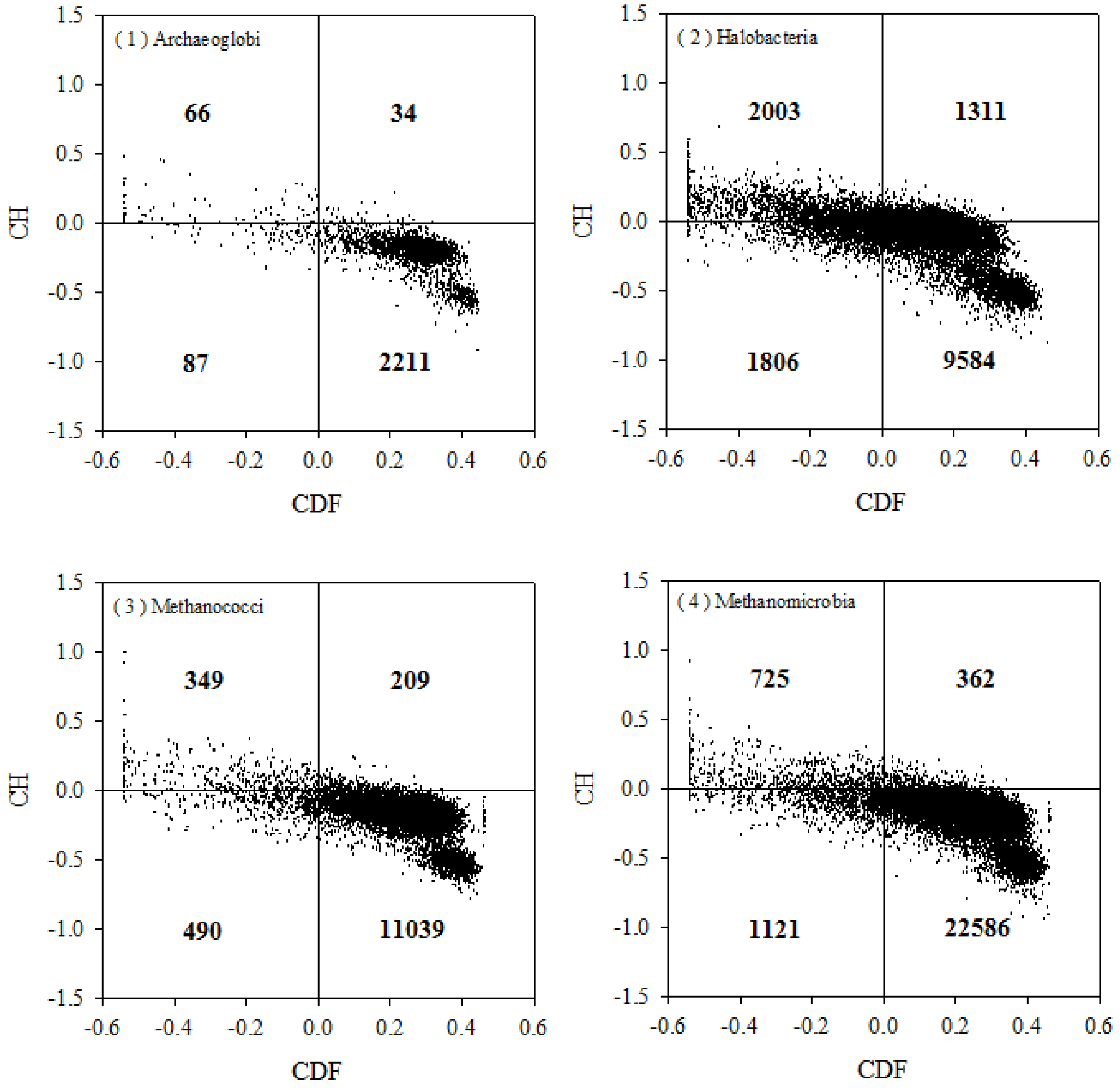

Figure S2. Comparison of the PONDR® VSL2 CDF and CH-plot analyses of whole protein order-disorder via distributions of proteins in the 8 **Euryarchaeota** classes within the CH-CDF phase space.

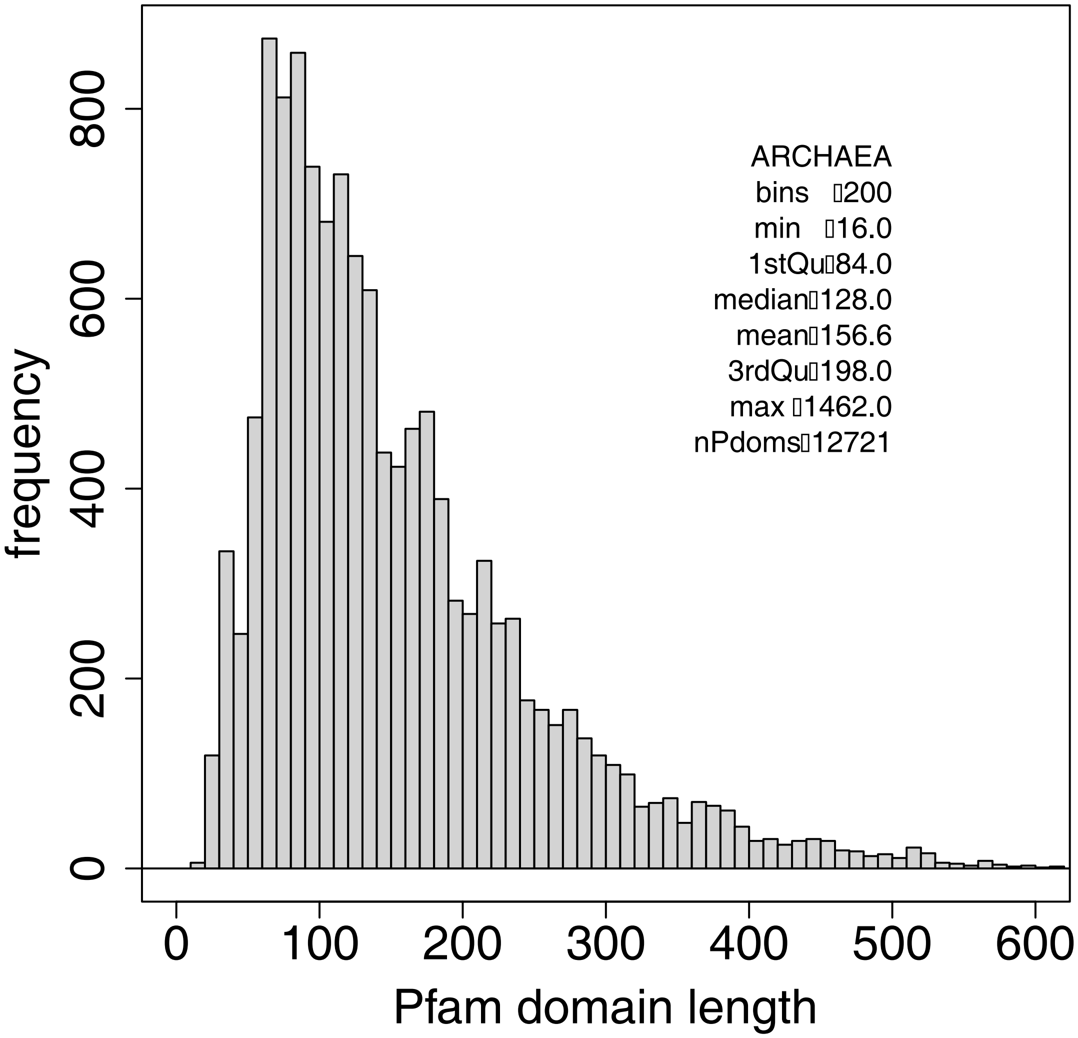

Figure S3. Length distribution of the 12,721 Pfam domain seeds of Archaean origin.

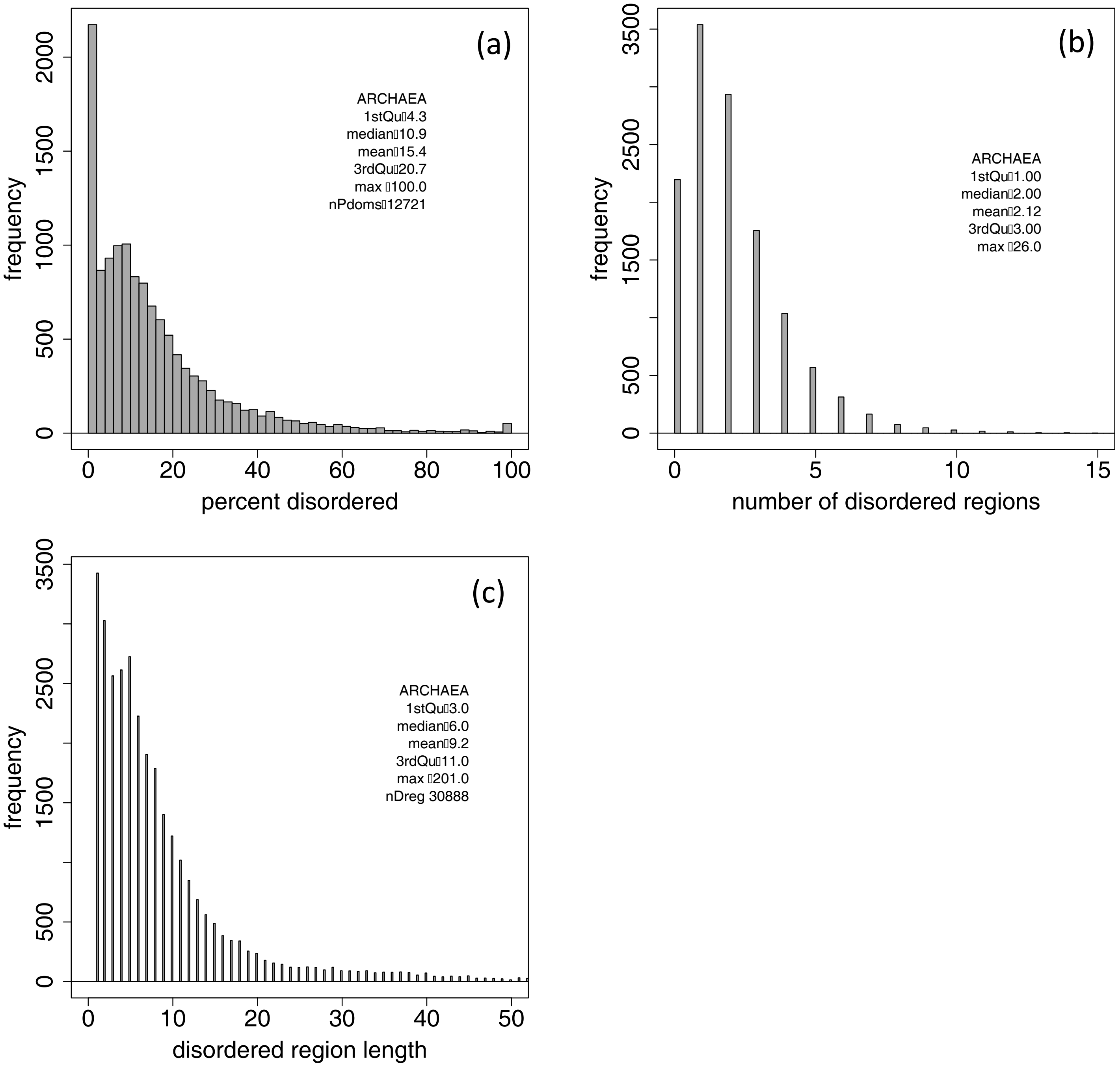

Figure S4. Abundance of intrinsic disorder in functional Pfam domains of the Archaean origin. (a) Frequencies of variously disordered domains; (b) Abundance of intrinsically disordered regions in various domains; and (c) the length distribution of intrinsically disordered regions found in Archaea Pfam domains.

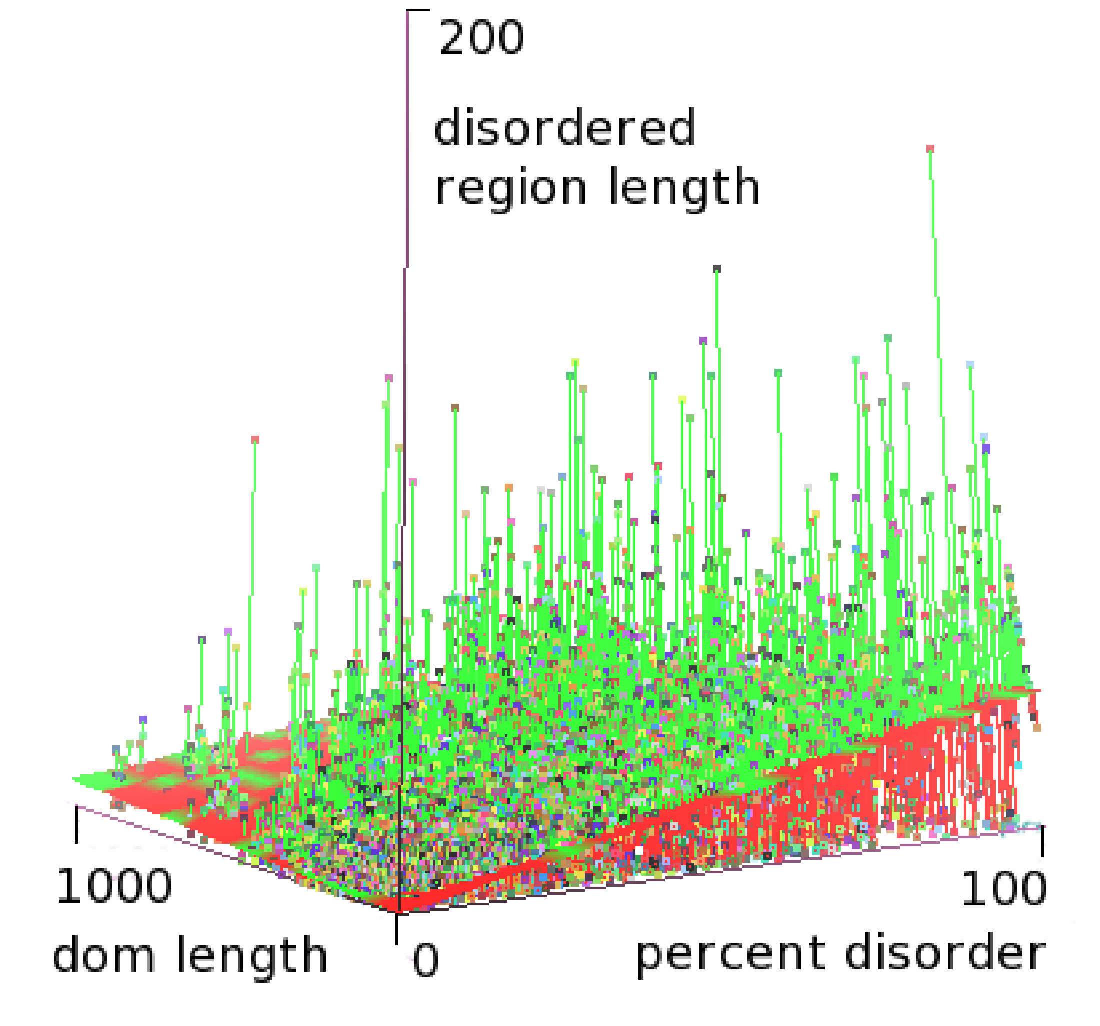

Figure S5. A three dimensional plot of total percent disorder, disordered region length (where there are up to 26 disordered regions per domain), and domain length for all Archaea seed domains in version 23.0 of the Pfam database.

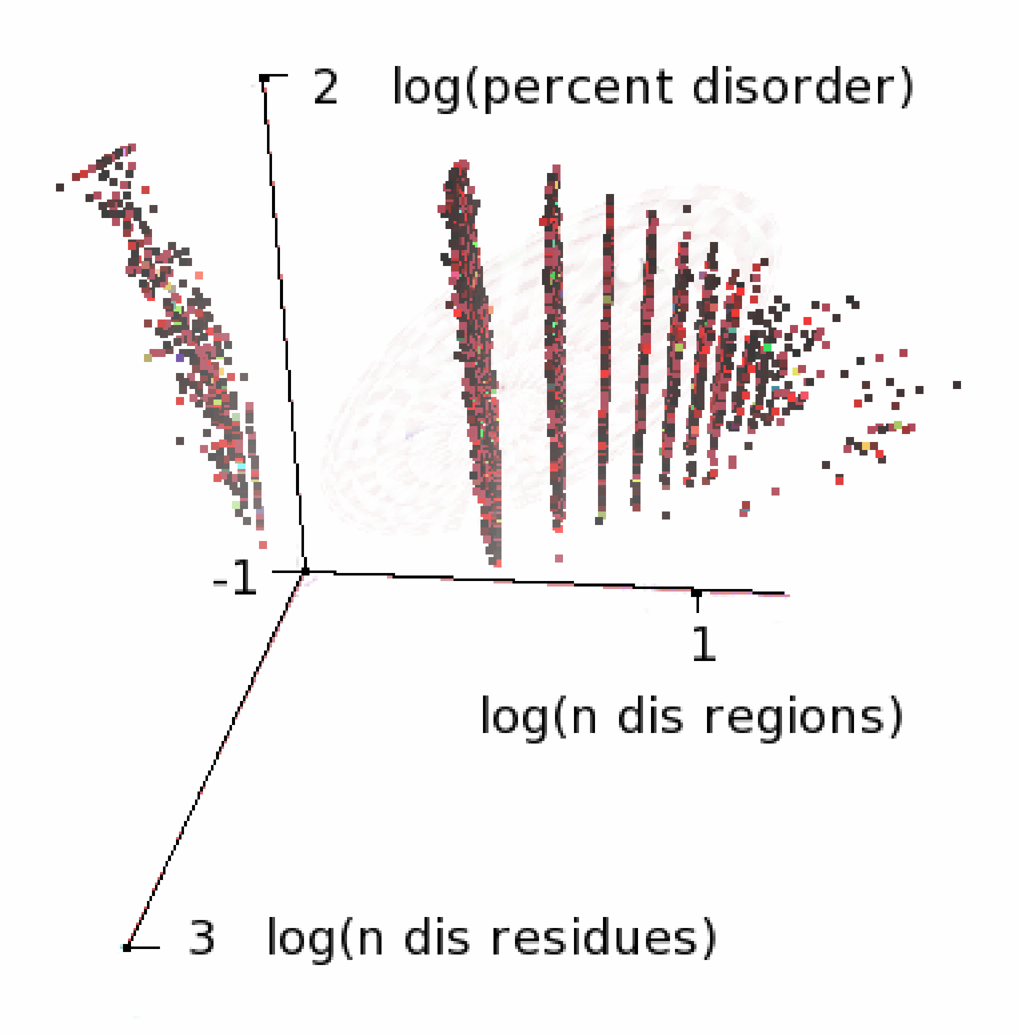

Figure S6. A three dimensional plot of the log of the number of disordered regions, the log of the number of disordered residues, and the log of the percent disorder in each of all of the Archaea seed domains in version 23.0 of the Pfam database.
